# Adrenal gland dysfunction in males with cluster headache

**DOI:** 10.1186/s10194-026-02386-z

**Published:** 2026-05-13

**Authors:** Connar Stanley James Westgate, Tanja Lylloff, David Møbjerg Kristensen, Thomas Folkmann-Hansen, Nunu Lund, Mads Barloese, Marie-Louise Kulas Søborg, Sophie Bryde Laursen, Trine Holm Johannsen, Hanne Frederiksen, Anders Juul, Rigmor Højland Jensen, Anja Sofie Petersen

**Affiliations:** 1 Danish Headache Center, Department of Neurology, Rigshospitalet-Glostrup, University of Copenhagen, Glostrup, Denmark; 2 Translational Research Centre Rigshospitalet, Glostrup, Denmark; 3 Health Technology, Danish Technical University, Lyngby, Denmark; 4 Department of Growth and Reproduction, Copenhagen University Hospital— Rigshospitalet, Copenhagen, Denmark; 5 University of Rennes, Inserm EHESP, Irset (Institut de Recherche en Santé, Environnement et Travail)-UMR_S 1085, Rennes, France; 6 Department of Science and Environment, Roskilde University, Roskilde, Denmark; 7 Danish Multiple Sclerosis Center, Department of Neurology, Glostrup, Rigshospitalet-Glostrup, University of Copenhagen, Copenhagen, Denmark; 8 Department of Radiology and Nuclear Medicine, Copenhagen University Hospital Hvidovre, Copenhagen, Denmark; 9 Department of Clinical Physiology and Nuclear Medicine, Centre for Functional and Diagnostic Imaging and Research, University of Copenhagen, Hvidovre Hospital, Hvidovre, Denmark; 10 Department of Clinical Medicine, Faculty of Health and Medical Sciences, University of Copenhagen, Copenhagen, Denmark; 11 International Centre for Research and Research Training in Endocrine Disruption of Male Reproduction and Child Health, Copenhagen University Hospital— Rigshospitalet, Copenhagen, Denmark; 12 Department of Clinical Biochemistry, Copenhagen University Hospital – Rigshospitalet, Copenhagen, Denmark

## Abstract

**Background:**

Cluster headache is associated with compensated hypogonadism in males, suggesting impaired testicular steroidogenesis. It is unknown if adrenal steroidogenesis is dysregulated and how this is linked to cluster headache pathophysiology. We therefore aimed to define the adrenal steroid profile in cluster headache and define the differences between the phases of episodic cluster headache. We secondarily aimed to assess whether the steroid profile could distinguish between chronic cluster headache and episodic cluster headache.

**Methods:**

A prospective case-control study containing adult males with chronic cluster headache (*n* = 60), paired episodic cluster headache in and out of bout (*n* = 60) and healthy controls (*n* = 60). A fasted serum steroid profile for 16 steroid hormones was assessed via liquid chromatography tandem mass-spectrometry, assessing mineralocorticoids, glucocorticoids, and androgens. Explorative machine learning was used to segregate the cluster headache states.

**Results:**

The mineralocorticoids 11-deoxycorticosterone (*P* < 0.05), corticosterone (*P* < 0.05) and aldosterone (*P* < 0.05) were lower in those with episodic cluster headache compared to controls. 11-deoxycorticosterone (*P* < 0.01), corticosterone (*P* < 0.01), and the glucocorticoids 11-deoxycortisol (*P* < 0.01) and cortisol (*P* < 0.05) and cortisol/DHEAS ratio (*P* < 0.001) are lower in episodic in bout compared to their paired remission state. Those with chronic cluster headache had lower concentrations of 17-OH pregnenolone (*P* < 0.05) and DHEAS (*P* < 0.001) and a higher cortisol/DHEAS ratio (*P* < 0.01). The steroid hormone profile distinguished between chronic and episodic cluster in bout with a specificity of 86% (95%CI: 69%-90%), sensitivity of 81% (95%CI: 73%-93%) and AUC of 0.92 (*P* < 0.0001). Attack frequency and time from last attack correlated with the degree of steroid dysfunction in episodic cluster headache but not in chronic cluster headache.

**Conclusion:**

We identify distinct steroid hormone profiles in episodic and chronic cluster headache. Episodic in bout was associated with reduced adrenal steroid synthesis that resolves in remission. Chronic cluster headache was associated with stress induced androgen suppression. Together, these differences are likely secondary to cluster headache and may reflect distinct neurobiological bases underlying episodic and chronic cluster headache.

**Clinical trail number:**

Not applicable.

**Graphical Abstract:**

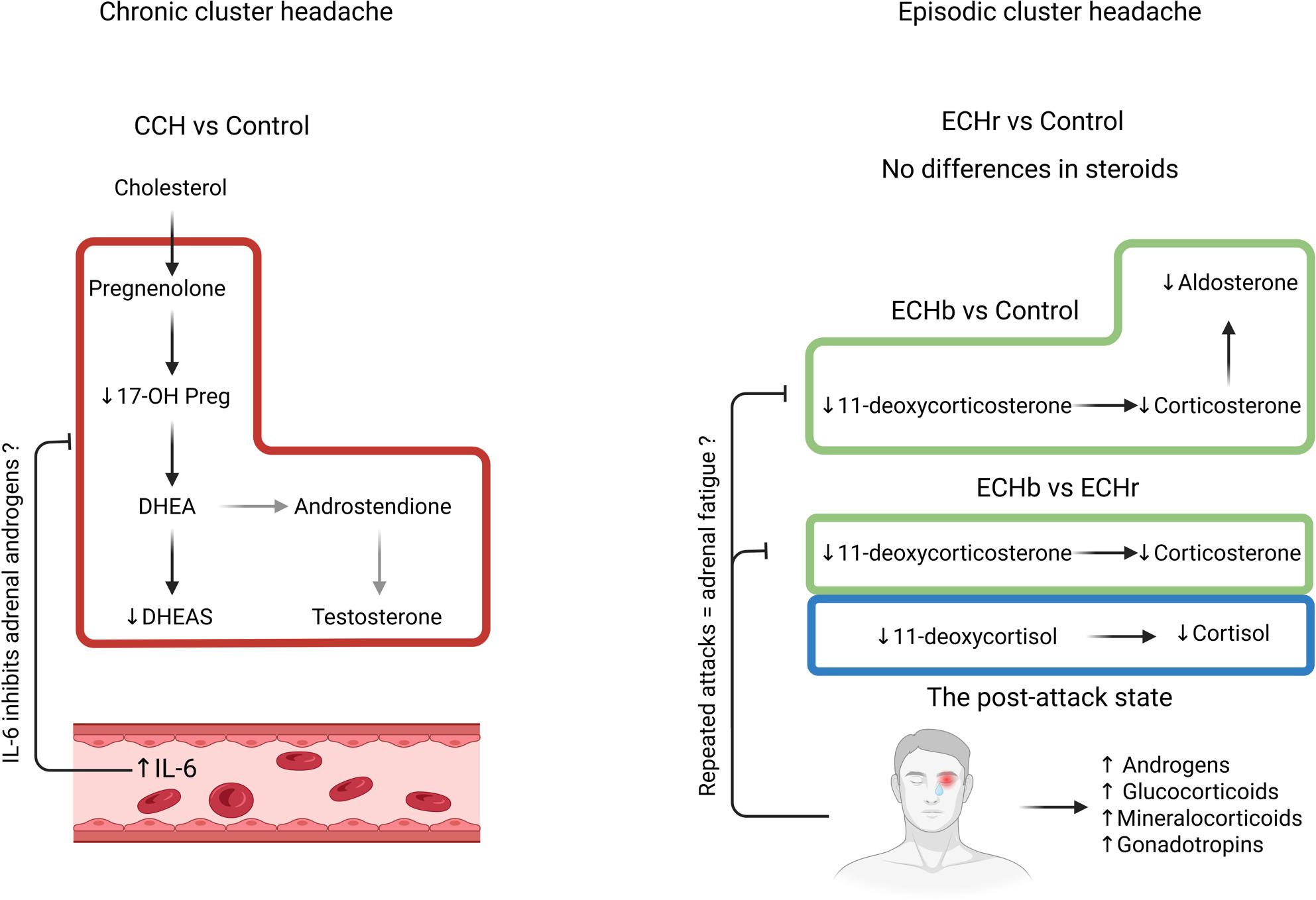

**Supplementary Information:**

The online version contains supplementary material available at 10.1186/s10194-026-02386-z.

## Background

Cluster headache (CH) is a primary headache disorder, defined by periods of intense unilateral orbital and/or periorbital pain, and is considered among the most severe pains a human can experience [1]. These attacks, 15–180 min in length, occur up to eight times per day and are associated with ipsilateral autonomic symptoms and restlessness [1]. Individuals experiencing attack-free periods longer than three months within the preceding year are classified as having episodic cluster headache (ECH), where those in the headache period are termed ECH in bout (ECHb) and those in a remission period are termed ECH in remission (ECHr). Those without these remission periods are classified as having chronic cluster headache (CCH) [1].

Cluster headache predominantly presents in males, with a male-to-female ratio of 4 − 3:1, which contrasts with other headache disorders that are more prevalent in females [2]. This suggests that sex steroids could play a role in cluster headache. Indeed, previous studies have demonstrated reduced concentrations of free testosterone and compensated hypogonadism in males with cluster headache [3]. Gonadal androgens are regulated by the hypothalamic-pituitary-gonadal (HPG) axis via the action of luteinizing hormone (LH), suggesting impaired hypothalamic signalling. The hypothalamus secretes releasing hormones that cause the release of the pituitary releasing hormones, follicle-stimulating hormone (FSH), LH and adrenocorticotropic hormone (ACTH), where impaired hypothalamic-pituitary signalling has been suggested in ECH [4–6]. This suspected hypothalamic dysfunction further suggests that the other steroid producing organ, the adrenal gland, could also be dysregulated in cluster headache [1].

The adrenal gland synthesizes all steroid classes and contributes to the circulating steroid milieu (Supplemental Fig. 1). The adrenal gland synthesizes androgens in the zona reticularis under the influence of ACTH. The major adrenal androgen is dehydroepiandrosterone-sulphate (DHEAS), with its corresponding precursors dehydroepiandrosterone (DHEA) and 17-OH pregnenolone, and is a circulating precursor for the androgens. Androstenedione and testosterone are minor adrenal androgens, where the gonads produce the majority of their circulating levels.

The adrenal glands exclusively synthesize the mineralocorticoids and glucocorticoids. The mineralocorticoids are synthesized in the zona glomerulosa and under the influence of angiotensin II and ACTH, which are modulators of blood pressure and fluid homeostasis, where aldosterone is the primary mineralocorticoid [4]. The mineralocorticoids 11-deoxycorticosterone and corticosterone are sequential biosynthetic precursors to aldosterone and have limited biological activity in humans. Glucocorticoids, of which cortisol is the primary human glucocorticoid, are regulators of cellular metabolism and act as the peripheral effectors of the hypothalamic circadian rhythm via the hypothalamic-pituitary-adrenal axis (HPA) [4]. Cortisol is synthesized in the zona fasciculata, where 11-deoxycortisol is the adrenal precursor. Cortisol is inactivated in the kidneys to cortisone, the inactive circulating glucocorticoid. Cortisol, also known as the ‘stress’ hormone, has been the subject of study in cluster headache, with reports demonstrating both higher or unchanged cortisol levels compared to controls [7]. However, these studies included low numbers of cases and controls, only assessed ECH in both periods, and measured cortisol alone [5–10].

These limitations hinder interpretation of the potential changes in cortisol and leave the origin of these alterations unclear. To address these gaps, comprehensive steroid profiling in cluster headache is warranted to define the circulating steroid hormone profile.

In this study, we hypothesised that steroid hormone profiles differ across cluster headache states. We therefore aimed to (i) define the steroid profile of the three cluster headache states in males compared with controls using gold-standard liquid-chromatography tandem mass-spectrometry, (ii) determine differences between ECHb and ECHr, and (iii) assess the discriminative capacity of the steroid profile to differentiate CCH from ECHb.

## Methods

### Study design and setting

Data was obtained from the **Danish Cluster Headache Biobank**, a prospective case–control study conducted between 2018 and 2021 at the Danish Headache Center, Rigshospitalet-Glostrup, Denmark. Concentrations of testosterone, DHEAS, androstenedione, and 17-hydroxyprogesterone as well as the gonadotropins LH and FSH have previously been reported in males of this cohort, comparing healthy controls with different cluster headache states [3]. The remaining steroids included in the present study were measured concurrently but were not analysed nor presented in the earlier publication. The current analyses therefore utilised the same cohort. The study methodology has been published in detail previously and is summarised briefly below [3]. In compliance with the Helsinki Declaration, all participants provided written informed consent. The study was approved by the Capital Region Regional Health Research Ethics Committee (H-16048941) and the Danish Data Protection Agency.

### Participants

The inclusion and exclusion criteria for the Danish cluster headache biobank have previously been published, and the study-specific criteria were as follows: an ICHD-3 diagnosis of cluster headache for cases; or absence of cluster headache for controls [11]. Participants were fasted for a minimum of 8 h. Participants were excluded if they had chronic headaches (except cluster headache), known drug abuse or serious somatic or psychiatric disorders. All participants were required to be male and have a body mass index (BMI) below 30 kg/m^2^. Participants were also excluded if they used oral steroids, had received a greater occipital nerve block within the previous 30 days prior to inclusion or had received any other steroid modifying medication. ECHb was defined as having at least one cluster headache attack in the last week and ECHr was defined as at least 30 days without a cluster headache attack. Participants with cluster headache in their first bout were followed up to 1 year to establish the correct ICHD-3 diagnosis.

For controls, additional exclusion criteria were: a history of primary or secondary headaches (except infrequent tension type headache), acute headache caused by alcohol, headaches associated with infections, having a 1st or 2nd degree relative with cluster headache, and having any headache in the past 7 days.

Participants with ECH had two study days: the first study day could occur either during a bout or during remission, and the second study day occurred in the opposite state. Controls and participants with established CCH had one study day.

### Outcomes

The primary outcomes were the differences in serum steroid concentrations between controls and the three cluster headache states: CCH, ECHb and ECHr. The main secondary end point was the capacity of steroid hormones to distinguish between CCH and ECHb. Further secondary endpoints included age-adjusted z-scores for steroid hormones and correlations of the steroid hormones and the gonadotropins LH and FSH to time from last cluster attack and the frequency of cluster attacks.

### Data sources

Blood was taken in 9-ml serum clot activator tubes (VACUETTE^®^) from an antecubital vein and inverted several times. Tubes rested at room temperature for 30 min prior to 10-minute centrifugation at 1409 g at 4 °C. Serum was subsequently aliquoted in polypropylene tubes (Greiner Bio-one) and initially stored at -25 °C prior to long term storage at -80 °C. Samples were sorted on dry ice to prevent freeze-thaw degradation.

Steroid analysis was performed using isotope diluted on-line Turbo-Flow LC-MS/MS [12]. The steroids quantified were the progestogens progesterone, 17-OH pregnenolone, 17-OH progesterone and progesterone; the mineralocorticoids 11-deoxycorticosterone, corticosterone and aldosterone; the glucocorticoids 11-deoxycortisol, cortisol and cortisone; the androgens dehydroepiandrosterone (DHEA), DHEAS, androstenedione, testosterone and dihydrotestosterone; and the oestrogen estrone-1-sulphate. These steroids represent the active circulating serum steroid hormones, as well as their primary circulating biosynthetic precursors. Estrone-1-sulphate represents the circulating store of oestrogens. The lower limits of detection for each steroid can be found in supplementary Table 1. In addition to the absolute steroid values, the derived measurements of the cortisol/cortisone ratio as a measurement of systemic glucocorticoid activity, and cortisol/DHEAS ratio as a measurement of systemic stress and adrenal function were calculated and utilized [13, 14]. Steroid values below the lower limit of detection (LOD) were excluded from analysis, no imputation was used. Age-adjusted z-scores for steroids were derived from age-related reference intervals based on a large Danish healthy reference cohort, where the population average is 0 [12].

The concentrations of LH and FSH were determined by chemiluminescence immunoassays (Atellica, Siemens Healthineers, Tarrytown, NY, USA) with LODs of 0.07 IU/L and 0.3 IU/L, respectively. All analytical methods used were accredited according to the ISO-standard 15,189:2013, DANAK registration number 1013. Previously published data on the inflammatory cytokine interleukin-6 in this cluster headache cohort, determined by O-link, were used in this study [15].

Clinical data was obtained through a semi-structured interview.

### Bias

Berkson and selection bias was reduced through inclusion of individuals with cluster headache who contacted the Danish Headache Centre (DHC) for guidance or oxygen treatment. Patients did not need to be followed at the DHC prior to inclusion and participants with other severe illnesses were excluded. Hospital control bias was avoided through recruiting healthy controls outside hospital settings.

### Missing data

Where steroid concentrations were below the LOD, the individual data point was excluded from the analysis. Steroid data was not imputed for direct comparisons and correlations but was imputed for machine learning. For aldosterone the number of samples in each group were: *N* = 40 for control, *N* = 51 for CCH, *N* = 36 for ECHb and *N* = 48 for ECHr. For DHEA the number of samples for each group were: *N* = 58 for control, *N* = 48 for CCH, *N* = 54 for ECHb and *N* = 58 for ECHr. 21-deoxycortisol (21DOC) was excluded from analysis due to a high degree of missingness.

### Machine learning

Machine learning was used to exploratorily distinguish between different cluster headache states. To meet the assumptions for the analysis, missing data was imputed using the median, and values were quantile normalized. Data for 21DOC was excluded due to a high percentage of missingness. A seed of 23 was used in the analysis.

To identify the most influential features for classification, a Random Forest model was trained using the randomForest function in R (version 4.5.0) with steroid hormones used as the predictors. The random forest model was implemented using default settings: ntree = 500, mtry = sqrt (p), nodesize = 1 and replace =TRUE. The importance of each feature was extracted from the model using the importance function, which provides a ranking based on the Mean Decrease Accuracy metric. Features were then sorted in descending order based on their importance scores and were used for the subsequent modelling steps.

A logistic regression model was trained to predict the binary outcome variable using the features identified in the feature selection process. The model was developed using the caret package in R, utilizing a generalized linear model (GLM) with a binomial family to handle the binary classification. To account for model variability and ensure robust performance, a 5-fold repeated stratified cross-validation approach was applied. Specifically, 5-fold cross-validation was repeated 10 times, with stratification to maintain the proportion of the target classes in each fold. The trainControl function was used to specify the cross-validation parameters, including saving all predictions and optimizing the model according to the ROC metric.

Model performance was evaluated using the area under the receiver operating characteristic (ROC) curve (AUC). The predicted probabilities for the positive class were obtained from the GLM. An ROC curve was generated using the roc function from the pROC package in R, and the AUC was calculated to assess the discriminatory power of the model. The AUC value provides a measure of the model’s ability to distinguish between the two classes, with values closer to 1 indicating better performance. A diagnostic odds ratio was calculated.

### Statistics

To ensure that the data met the assumptions for parametric testing, all statistics on steroid data were performed on Log_10_ transformed data. Comparisons between the cluster headache states and controls were performed using one-way ANOVA followed by post-hoc Dunnett’s tests to correct for multiple comparisons within each steroid. Linear regression models were used to compare the cluster headache states to healthy controls while taking co-variates into account. Here, Log_10_ transformed steroid hormone concentration was the dependent variable, age and timepoint of blood sampling were included as continuous variables. For paired comparisons between bout and remission states, paired t-tests were performed. Pearsons’s correlation was performed for correlation analysis. Statistical analysis was performed in R (version 4.5.0). A p-value of < 0.05 was considered statistically significant.

## Results

### Demographics

Among the participants in the Danish Cluster Headache Biobank, 60 controls, 60 with CCH, and 60 with ECH met the initial inclusion criteria, resulting in 59 pairs in the ECH group (Table 1). All participants were males, and the age at inclusion was 45.9 (Min:22, Max:65) years for CCH, 41.6 (Min:22, Max:76) years for ECH and 41.3 (Min:22, Max:64) years for controls. After steroid quantification, three individuals were considered outliers. Here, two reported exogenous androgen supplementation and one required further clinical follow-up for pathology. This resulted in 56 pairs of ECH, with 58 individuals in ECHb, 59 individuals in ECHr and 59 with CCH for the final analysis.

On the day of sampling, 35 participants (60%) with ECHb and 38 (64%) participants with CCH had an attack within the previous 24 h. Table 2 summarises the clinical variables on the day of sampling.

**Table 1:** Demographics on initial inclusion

	Healthy controls (*n* = 60)	Chronic Cluster Headache (*n* = 60)	Episodic Cluster Headache (*n* = 60)
Age in years, mean (Min-Max)	Age in years, mean (Min-Max)	45.9 (23–76)	41.6 (22–64)
BMI, mean (SD)	-	26.1 (18.2–29.8)	25.1 (17.9–29.7)
CH history in years, median (IQR)	-	14 [8; 21.5]	13.5 [6; 20]
Average attack duration in minutes, median (IQR)	-	83 [60;125]	60 [50; 120]
Pain intensity (0–10, NRS), median (IQR)	-	10 [10; 10]	10 [8; 10]
Attacks per day, mean (Min-Max)	-	2.4 (0–6)	2.1 (0–7)
Smoking			
Current	6 (10%)	35 (58%)	39 (65%)
Prior	14 (23%)	11 (18%)	16 (27%)
Never	40 (67%)	14 (23%)	5 (8%)

**Table 2:** Day of sampling parameters

Parameter	Controls (*n* = 60)	CCH (*n* = 59)	ECHb (*n* = 58)	ECHr (*n* = 59)
Cluster attacks past 24 h	-	1 [0;3]	1 [0;2]	
Time since last attack				
< 2 h	-	6 (10%)	4 (7%)	-
2 to < 6 h	-	13 (22%)	13 (22%)	-
6 to < 12	-	11 (19%)	8 (14%)	-
24 h	-	8 (14%)	7 (12%)	-
> 24 h	-	21 (36%)	23 (40%)	-
Missing	-	0	3 (5%)	-
Cluster attacks last week	-	10 [2;20]	10 [5;14]	-
Preventatives				
Verapamil	-	26 (44%)	20 (34%)	7 (12%)
Another preventative	-	10 (16%)	2 (3%)	4 (7%)
Acute medication				
Oxygen	-	21 (36%)	18 (31%)	-
Triptans	-	5 (8%)	15 (26%)	-
NSAID	0 (0%)	4 (7%)	6 (10%)	2 (3%)
Paracetamol	0 (0%)	3 (5%)	1 (2%)	0
Opioids	0 (0%)	2 (3%)	1 (2%)	1
Other medications				
Sartan	0	0	0	1(2%)
ACE inhibitors	1	1 (2%)	0	0
Time of blood sampling				
6:00–8:59	38 (63%)	19 (32%)	15 (26%)	30 (50%)
9:00–10:59	20 (33%)	22 (37%)	28 (48%)	21 (39%)
11:00–12:59	1 (2%)	13 (22%)	10 (17%)	0 (0%)
13:00–14:00	1 (2%)	5 (8%)	4 (7%)	2 (3%)

Data presented as median [IQR] or count (%)

### Controls vs. ECH

The profiles of 15 unadjusted steroid hormones and derivative ratios in males with cluster headache and healthy male controls are shown in Table 3. A whole steroid profile for age and time of sample adjusted steroids can be found in supplemental Fig. 2 and the model can be found in supplemental Table 2.

**Table 3:** Unadjusted steroid levels in controls, chronic cluster headache (CCH), episodic cluster headache in bout (ECHb) and episodic cluster headache in remission (ECHr)

Steroid	Control (*n* = 60)	CCH (*n* = 59)	ECHb (*n* = 58)	ECHr (*n* = 59)
Progestogens (nmol/l)				
Progesterone	0.36 (0.08)	0.35 (0.11)	0.37 (0.19)	0.38 (0.17)
17-OH Pregnenolone	10.05 (6.48)	7.05 (5.71)**	9.73 (9.15)	11.51 (8.81)
17-OH Progesterone	3.43 (1.34)	3.20 (1.17)	3.16 (1.21)	3.47 (1.47)
Mineralocorticoids (nmol/l)				
11-deoxycorticosterone	0.14 (0.07)	0.16 (0.08)	0.11 (0.05)*	0.16 (0.12)
Corticosterone	10.82 (6.73)	9.30 (6.81)	6.94 (4.66)*	12.37 (12.87)
Aldosterone^1^	0.31 (0.32)	0.32 (0.25)	0.19 (0.08)*	0.21 (0.15)
Glucocorticoids (nmol/l)				
11-deoxycortisol	1.06 (0.69)	1.26 (0.84)	0.95 (0.67)	1.30 (0.86)
Cortisol	349.2	313.8 (101.2)	308.6 (96.8)	361.4 (12.4)
Cortisone	62.87 (14.20)	56.99 (15.64)	63.87 (15.09)	69. 01 (15.87)
Androgens (nmol/l)				
Dehydroepiandrosterone^2^	17.16 (10.46)	14.05 (8.07)	14.62 (9.09)	17.45 (9.55)
DHEAS	5767 (2785)	4095 (3190)****	5597 (2777)	5295 (2940)
Androstenedione	3.39 (1.03)	3.65 (1.66)	3.51 (1.18)	3.89 (1.29)
Testosterone	20.84 (6.46)	18.52 (6.04)	19.32 (6.45)	19.85 (7.55)
Dihydrotestosterone	1.82 (0.73)	1.73 (0.67)	1.64 (0.66)	1.70 (0.60)
Oestrogen (nmol/l)				
Estrone-1-sulphate	2.31 (1.25)	1.90 (1.37)	1.72 (0.98)*	1.77 (1.02)
Derivative measurements				
Cortisol/cortisone	5.62 (1.48)	5.61 (1.43)	5.06 (1.16)	5.32 (1.67)
Cortisol/DHEAS	0.077 (0.049)	0.133 (0.115)**	0.067 (0.035)	0.086 (0.046)

Statistics performed on log_10_ steroid values. Data presented as mean ± SD *=*P* < 0.05, **=*P* < 0.01 and ****=*P* < 0.0001. Statistical comparisons relative to controls. ^1^ Due to values below lower limit of detection, number for each group in aldosterone: *N* = 40 for control, *N* = 51 for CCH, *N* = 36 for ECHb and *N* = 48 for ECHr. ^2^ Due to values below the lower limit of detection, number for each group in DHEA: *N* = 58 for control, *N* = 48 for CCH, *N* = 54 for ECHb and *N* = 58 for ECHr. Abbreviations: OH: hydroxy; DHEA: dehydroepiandrosterone; DHEAS: DHEA sulphate

**Fig. 1:**
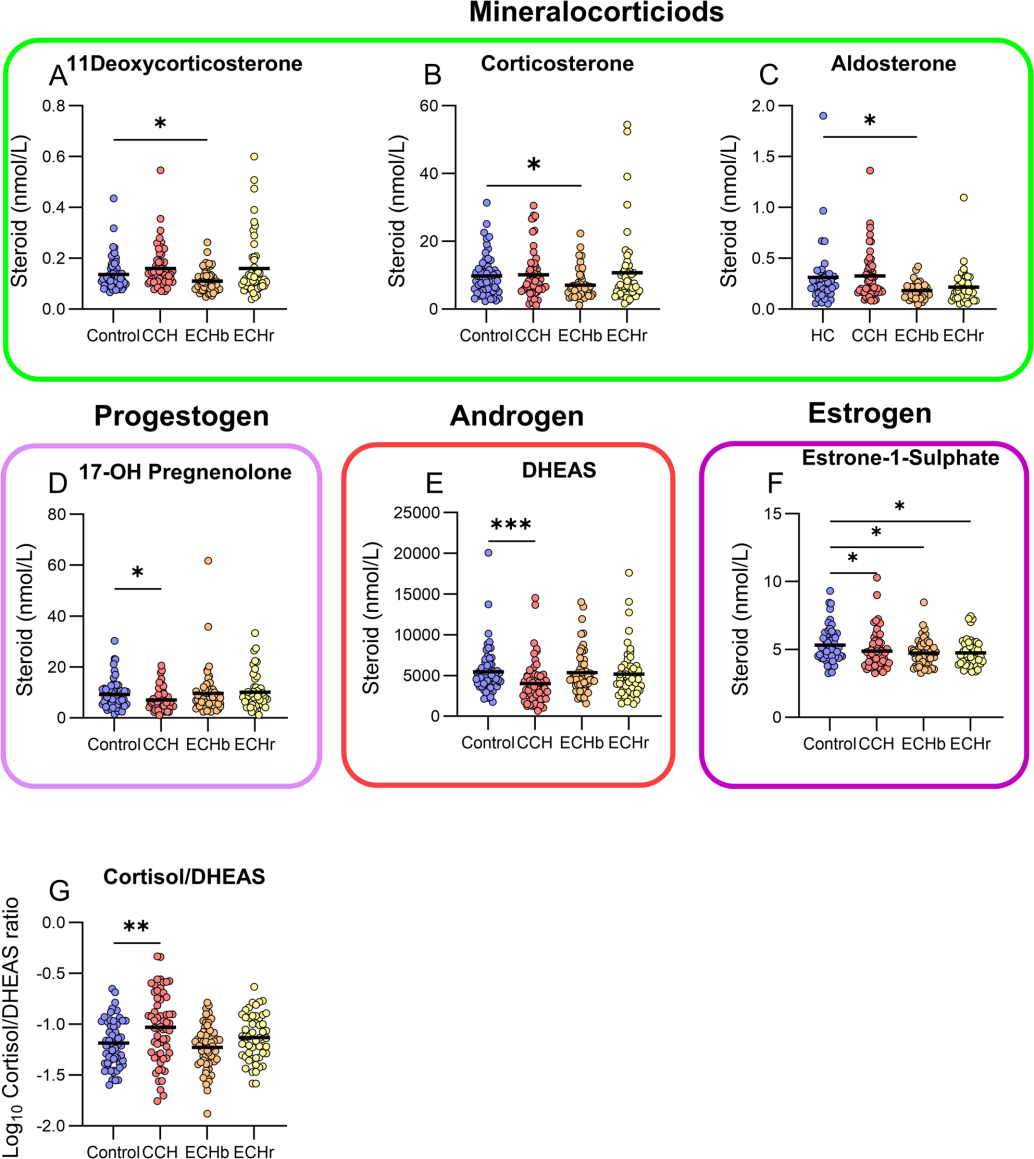
Steroids in cluster headache. Age and time of sampling adjusted serum steroid profiles in males with chronic cluster headache (CCH), episodic cluster headache in bout (ECHb), episodic cluster headache in remission (ECHr) and healthy male controls. Serum levels of **A**) 11-deoxycorticosterone, **B**) corticosterone, **C**) aldosterone, **D**) 17-OH pregnenolone, **E**) dehydroepiandrosterone-sulphate, **F**) estrone-1-sulphate and the **G**) cortisol/DHEAS ratio. One-way ANOVA followed by post hoc Dunnett’s test; statistics performed on Log_10_ transformed data. Control *n* = 60, CCH *n* = 59, ECHb *n* = 58 and ECHr *n* = 59. *=*P* < 0.05, **=*P* < 0.01 and ***=*P* < 0.001. Data presented as mean

In ECHb, after adjusting for age and time of sampling, we observed reductions in all mineralocorticoids, with reduced serum levels of 11-deoxycorticosterone (0.11 ± 0.04 vs. 0.14 ± 0.06 nmol/L, *P* = 0.03, Fig. 1A), corticosterone (7.09 ± 4.6 vs. 9.80 ± 5.97 nmol/L, *P* = 0.04, Fig. 1B), and aldosterone (0.18 ± 0.08 vs. 0.31 ± 0.32 nmol/L, *P* = 0.04, Fig. 1C) compared to controls. The oestrogen estrone-1-sulphate was lower in ECHb (4.73 ± 0.96 vs. 5.33 ± 1.26 nmol/L, *P* = 0.01, Fig. 1F). None of the progestogens, androgens, or glucocorticoids differed between ECHb and controls. The findings were consistent with the age adjusted z-scores (supplemental Fig. 3, supplemental Table 3).

In ECHr, no differences in steroid concentrations were found in comparison to controls, except for estrone-1-sulphate, which was lower (4.75 ± 0.99 vs. 5.33 ± 1.26 nmol/L, *P* = 0.02, Fig. 1F) than controls.

The findings were consistent with the age-adjusted z-scores (supplemental Fig. 3, supplemental Table 3).

### Controls vs. CCH

In CCH, the progestogen 17-OH pregnenolone was reduced (7.02 ± 4.36 vs. 9.21 ± 5.60 nmol/L, *P* = 0.03, Fig. 1D) compared to controls, while no other progestogens were altered. The androgen DHEAS was found to be lower in CCH (4011 ± 2694 vs. 5438 ± 2882 nmol/L, *P* < 0.0002, Fig. 1E), whereas no other androgens were differential in CCH. A marker of adrenal function, the cortisol/DHEAS ratio was higher in CCH (0.12 ± 0.10 vs. 0.07 ± 0.04, *P* = 0.0037, Fig. 1G) compared to controls. Estrone-1-sulphate was lower in CCH (4.88 ± 1.36 vs. 5.33 ± 1.26 nmol/L, *P* = 0.04, Fig. 1F) compared to controls. No differences were found in mineralocorticoids or glucocorticoids in CCH. The findings were consistent with z-scores (Supplemental Fig. 3, Supplemental Table 3).

The cytokine IL-6 is known to suppress adrenal androgen production, where IL-6 has previously been demonstrated to be increased in this CCH cohort [15,16]. Given this, we assessed the correlation between IL-6 and the adrenal androgens. Serum IL-6 inversely correlates with 17-OH pregnenolone (*r*=-0.40, *P* = 0.002), DHEA (*r*=-0.37, *P* = 0.005) and a near inverse correlation with DHEAS (*r*=-0.25, *P* = 0.05). There was a positive correlation with cortisol/DHEAS ratio (*r* = 0.29, *P* = 0.03).

### Paired episodic in bout vs. remission

**Fig. 2:**
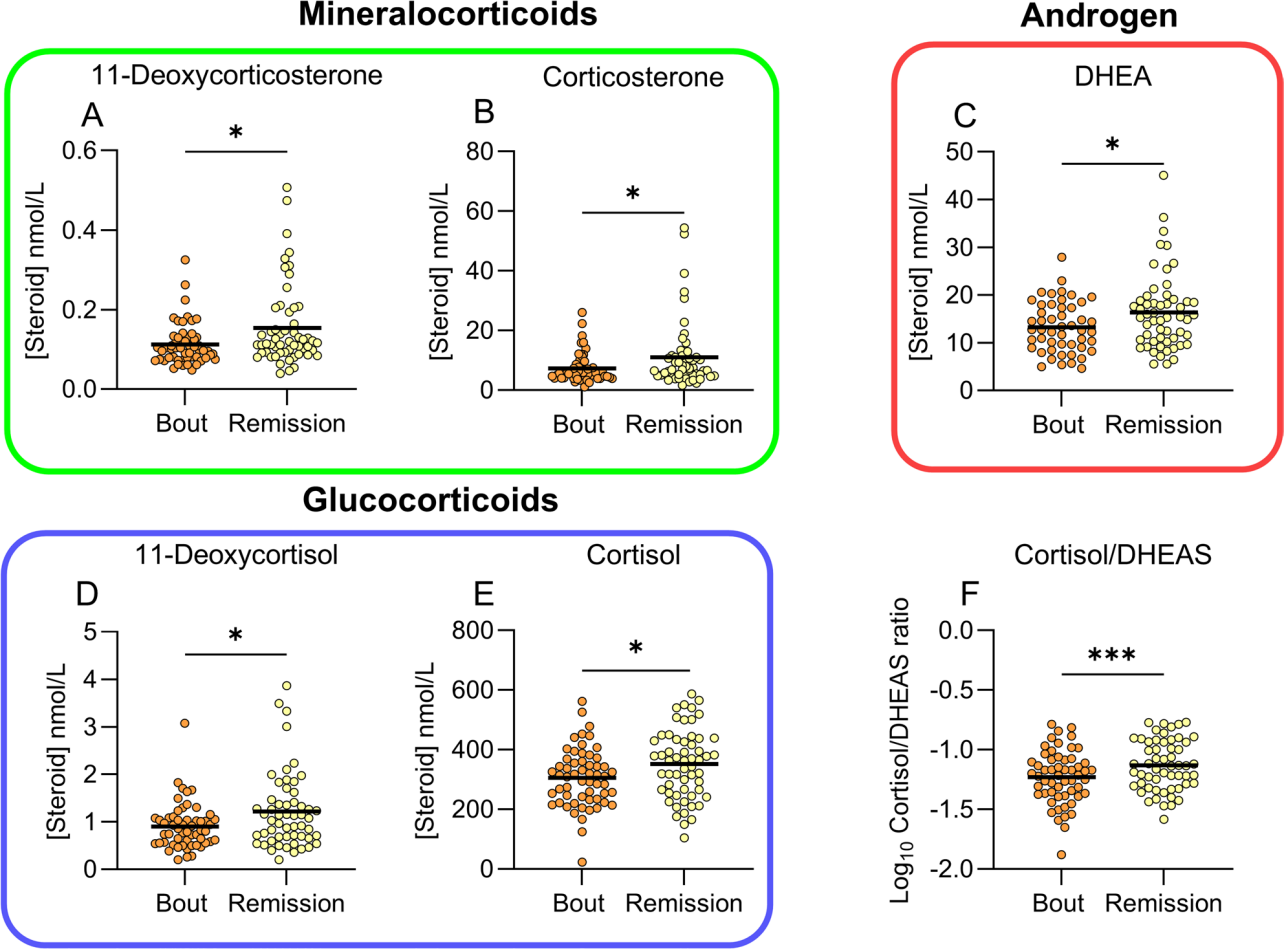
Steroids in paired episodic cluster headache. Age and time of sampling adjusted serum steroids in episodic cluster headache (*N* = 56), paired between bout and remission. Serum levels of **A**) 11-deoxycorticosterone, **B**) corticosterone, **C**) dehydroepiandrosterone, **D**) 11-deoxycortisol, **E**) cortisol and **F**) cortisol/DHEAS ratio. Paired t-tests on the log_10_ transformed data. *N* = 56 pairs. *=*P* < 0.05, and ***=*P* < 0.001. Data presented as mean

We also conducted an analysis on those 56 individuals with episodic cluster headache who had paired bout and remission blood samples, unadjusted values can be found in Table 4. After correcting for time of sampling and age 11-deoxycorticosterone (0.11 ± 0.05 vs. 0.15 ± 0.10 nmol/L, *P* = 0.011, Fig. 2A) and corticosterone (7.28 ± 4.88 vs. 11.01 ± 11.06 nmol/L, *P* = 0.04, Fig. 2B) were 26% and 38% lower respectively in ECHb compared to remission. Dehydroepiandrosterone was 23% lower (13.26 ± 5.31 vs. 17.35 ± 7.99 nmol/L, *P* = 0.04, Fig. 2C) in bout. Cortisol (305 ± 97 vs. 352 ± 116 nmol/L, *P* = 0.01, Fig. 2D) and 11-deoxycortisol (0.90 ± 0.48 vs. 1.22 ± 0.78 nmol/L, *P* = 0.02, Fig. 2F) were 13% and 26% lower respectively in bout. The cortisol/DHEAS ratio was 22% lower (0.066 ± 0.033 vs. 0.083 ± 0.040, *P* = 0.0008, Fig. 2F) in bout. All other steroids were unaltered.

**Table 4:** Unadjusted steroid levels in paired episodic in bout an remmsion

Steroid	ECHb (*n* = 56)	ECHr (*n* = 56)	Δ (%)	*P*-value
Progestogens (nmol/l)				
Progesterone	0.37 (0.19)	0.38 (0.18)	-0.01 (-3%)	0.59
17-OH Pregnenolone	9.78 (9.28)	11.47 (8.99)	-1.69 (-14%)	0.24
17-OH Progesterone	3.19 (1.21)	3.50 (1.46)	-0.31 (-9%)	0.13
Mineralocorticoids (nmol/l)				
11-deoxycorticosterone	0.11 (0.05)	0.14 (0.09)	-0.03 (-21%)	0.0045
Corticosterone	6.79 (4.59)	11.57 (12.10)	-4.78 (-41%)	0.0022
Aldosterone^1^	0.20 (0.09)	0.18 (0.09)	0.02 (11%)	0.25
Glucocorticoids (nmol/l)				
11-deoxycortisol	0.88 (0.47)	1.24 (0.80)	-0.36 (-29%)	0.0079
Cortisol	308.4 (105.23)	358.1 (122.11)	-49.7 (-14%)	0.04
Cortisone	63.8 (15.3)	68.5 (16.1)	-4.7 (-7%)	0.17
Androgens (nmol/l)				
Dehydroepiandrosterone^2^	14.48 (9.10)	17.36 (9.58)	-2.9 (-17%)	0.028
DHEAS	5618 (2807)	5205 (2759)	413 (8%)	0.07
Androstenedione	3.53 (1.18)	3.88 (1.32)	-0.35 (-9%)	0.09
Testosterone	18.92 (6.18)	19.96 (7.26)	-1.04 (-5%)	0.35
Dihydrotestosterone	1.61 (0.65)	1.71 (0.60)	-0.1 (-6%)	0.04
Oestrogen (nmol/l)				
Estrone-1-sulphate	1.74 (0.99)	1.79 (1.03)	-0.05 (-3%)	0.51
Derivative measurements (ratios)				
Cortisol/cortisone	5.04 (1.69)	5.31 (1.69)	-0.27 (-5%)	0.21
Cortisol/DHEAS	0.066 (0.035)	0.085 (0.043)	-0.019 (-22%)	0.0004

Statistics performed on log_10_ steroid values. Data presented as means(SD)

^1^ *N* = 28 due to values not meeting lower limit of detection

^2^ *N* = 52 due to values not meeting lower limit of detection. Abbreviations: OH: hydroxy; DHEAS: DHEA sulphate

### The proximity to a cluster attack and steroid levels

#### ECHb

**Fig. 3:**
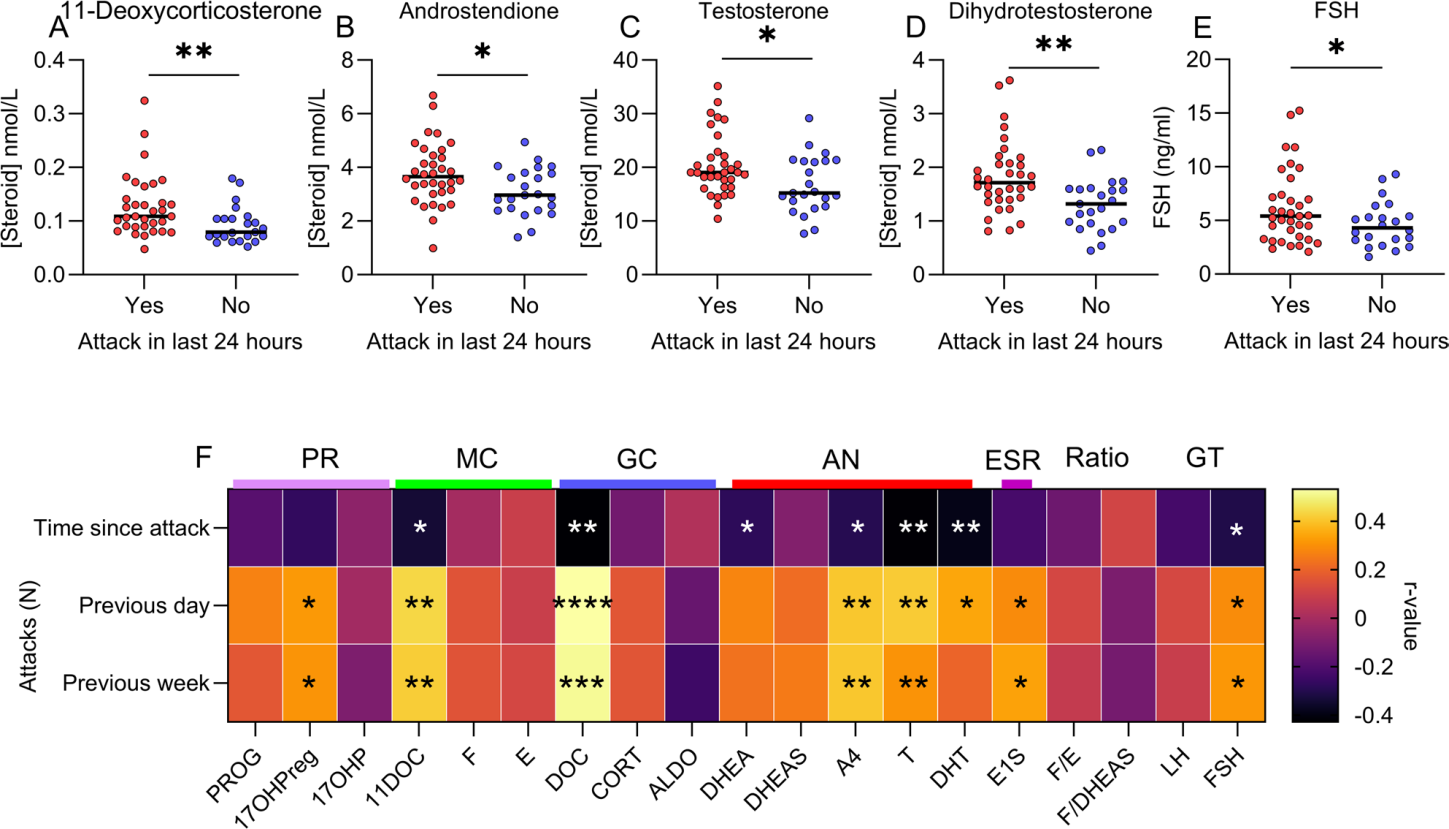
The effects of a cluster attack on the steroid profile. The relation of cluster attacks to age and time of sampling adjusted steroid levels in individuals with ECHb. **A**) The effect of having a cluster attack in the last 24-hours on the levels of **A**) 11-deoxycorticosterone, **B**) androstenedione, **C**) testosterone and **D**) dihydrotestosterone. The effect of having a cluster attack in the last 24-hours on the levels of **E**) follicle-stimulating hormone (FSH). **F**) A correlation matrix describing the relation of time since last cluster attack, number of attacks in the previous day and number of attacks in the previous week. T-tests for B, C, D, E, F and G. Statistical analysis performed in log_10_ transformed data for B, C, D and E. Pearson’s correlation on log_10_ transformed steroids and gonadotropins. Data presented as mean. *=*P* < 0.05, **=*P* < 0.01, ***=*P* < 0.001 and **** P = < 0.0001. Yes: *N* = 34, No: *N* = 23 Abbreviations: PROG = progesterone, 17OHPreg = 17-hydroxy pregnenolone, 17OHP = 17-hydroxy progesterone, DOC = 11-deoxycorticosterone, CORT = corticosterone, ALDO = aldosterone, 11DOC = 11-deoxycortisol, F = cortisol, E = cortisone, DHEA = dehydroepiandrosterone, DHEAS = dehydroepiandrosterone- sulphate, A4 = androstenedione, T = testosterone, DHT = dihydrotestosterone and E1S = estrone-1-sulphate, LH = luteinizing hormone, FSH = follicle-stimulating hormone, PR = progestogens, MC = mineralocorticoids, GC = glucocorticoids, AN = androgens, ESR = estrogen and GT = gonadotropin

In an exploratory analysis, we assessed whether having a cluster attack within the 24-hours prior to sampling affected steroid levels. In ECHb, higher levels of serum 11-deoxycorticosterone (38%, 0.13 ± 0.06 vs. 0.09 ± 0.03 nmol/L, *P* = 0.005, Fig. 3A), androstenedione (22%, 3.77 ± 1.64 vs. 3.10 ± 0.90 nmol/L, *P* = 0.04, Fig. 3B), testosterone (22%, 20.41 ± 5.7 vs. 16.73 ± 5.36 nmol/L, *P* = 0.012, Fig. 3C) and dihydrotestosterone (40%, 1.83 ± 0.67 vs. 1.31 ± 0.49 nmol/L, *P* = 0.0015, Fig. 3D) were found in those who had experienced an attack compared to those who had not experienced an attack in the 24 h prior to sampling. No other steroids were differential (Supplemental Table 4). Given that gonadotropins regulate serum androgen levels, we assessed whether gonadotropins were affected by attacks. FSH was higher in individuals with ECHb that had an attack in the 24 h prior to sampling compared to those who had not (36%, 6.21 ± 3.46 vs. 4.55 ± 2.08, *P* = 0.043, Fig. 3E). The levels of the corticosteroids 11-deoxycortisol and 11-deoxycorticosterone display an inverse correlation to time since last cluster attack, as do the androgens DHEA, androstenedione, testosterone, dihydrotestosterone, and the gonadotropin FSH (Fig. 3F, supplemental Table 4). In line with these results, the more resent a cluster attack, the higher the levels of the analytes.

Attack burden in terms of numbers of attacks in the last day and last week was also associated with steroid levels (Fig. 3F, supplemental Table 5). The corticosteroids 11-deoxycortisol and 11-deoxycorticosterone display a positive correlation to the number of attacks, as does the adrenal androgen precursor 17OH-pregnenolone. It follows that the androgens androstenedione, testosterone, and dihydrotestosterone are positively correlated with attack frequency. The downstream metabolite of androstenedione, estrone-1-sulphate also displays this association. The more attacks an individual had, the higher the level of these analytes in the blood.

### CCH

In contrast to ECH, no steroids are increased in those who had a cluster headache attack in the last 24 h (supplemental Table 6). Indeed, no association was found between steroid levels and the time since the previous attack (supplemental Table 7). In addition, the majority of steroid showed no association with attack burden, with only the cortisol/cortisone ratio and 11-deoxycortsiol showing a positive association with attack burden (supplemental Table 7).

### Clinical differentiation

**Fig. 4:**
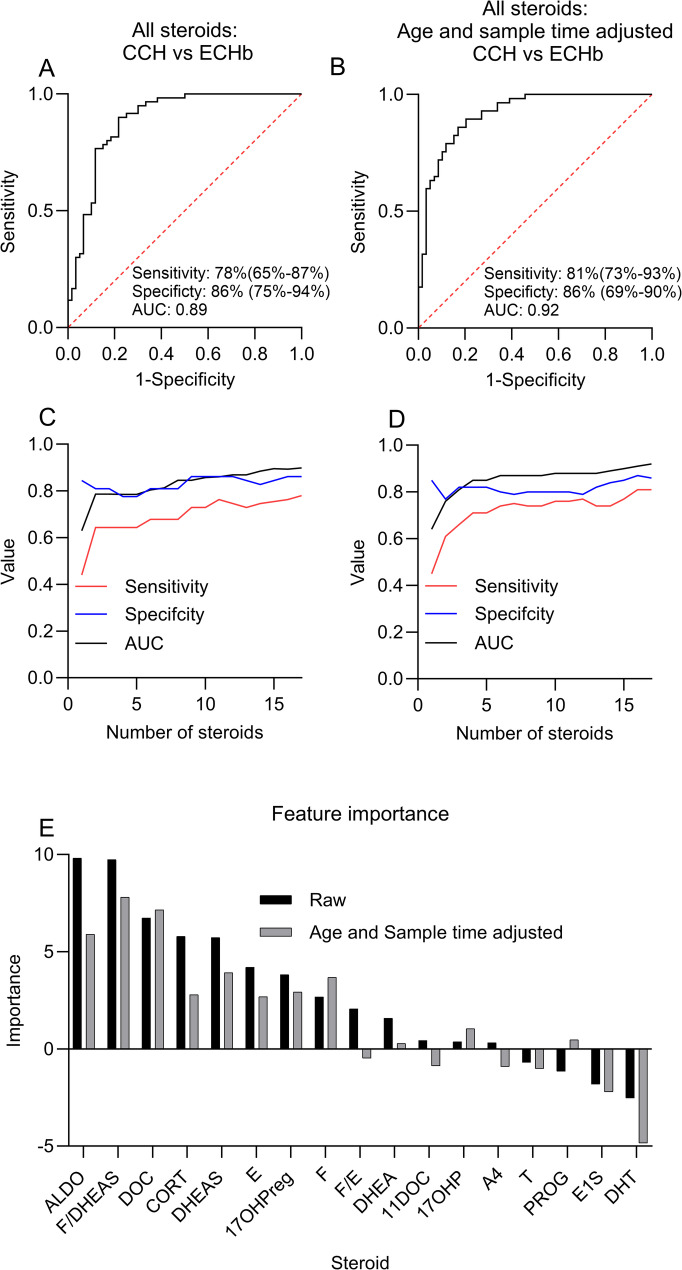
Biochemical distinction between episodic in bout and chronic cluster headache. Machine learning using random forest to distinguish between ECHb and CCH. A ROC curve for CCH vs. ECHb on **A**) raw steroids and age and **B**) sample time adjusted steroids, dotted line represents AUC 0.5. Model performance with different numbers of steroids with ascending importance for **C**) raw steroids and age and **D**) sample time adjusted steroids. **E**) A graph detailing the importance (unitless) of each steroid on the model. Abbreviations: PROG = progesterone, 17OHPreg = 17-hydroxy pregnenolone, 17OHP = 17-hydroxy progesterone, DOC = 11-deoxycorticosterone, CORT = corticosterone, ALDO = aldosterone, 11DOC = 11-deoxycortisol, F = cortisol, E = cortisone, DHEA = dehydroepiandrosterone, DHEAS = dehydroepiandrosterone- sulphate, A4 = androstenedione, T = testosterone, DHT = dihydrotestosterone and E1S = estrone-1-sulphate

We identified a distinct pattern of steroid dysregulation across the cluster headache states, characterized by lower corticosteroids in ECHb and adrenal androgen suppression in CCH, highlighting highly differential steroid profiles. Thus, we exploratorily assessed the ability of machine learning differentiate between CCH and ECHb based on steroid hormones. Using a random forest approach, ECHb and CCH could be distinguished with a sensitivity of 78% (95%CI: 65–87%) and a specificity of 86% (75–94%) with an AUC of 0.89 (Fig. 4A). The random forest algorithm shows significant discriminative performance, with an odds ratio of 22.1 (95%CI: 8.4–58.2%, *P* < 0.0001). This discriminative performance is marginally improved when adjusting values for age and time of sampling with a sensitivity of 81% (73–93%) and specificity of 86% (69–90%) with an AUC of 0.92 and an odds ratio of 26.7 (9.9–72.2%, *P* < 0.0001) (Fig. 4B). Evaluation of the number of steroids required for optimal performance indicated that the full steroid panel yielded the greatest discriminative power for both raw steroid values (Fig. 4C) and age and time of sampling adjusted values (Fig. 4D) The most important variables distinguishing CCH from ECHb were aldosterone and the cortisol/DHEAS ratio in the adjusted values, whereas the cortisol/DHEAS ratio and 11-doxycortisol were the most important distinguishing steroids (Fig. 4E).

## Discussion

This is the first study to comprehensively assess the steroid phenotype in individuals with cluster headache. By combining mass-spectrometry with explorative machine learning, we identified differential patterns of steroid dysregulation across the cluster headache states, characterized by reduced corticosteroids in ECHb and adrenal androgen suppression in CCH. Based on these distinct profiles, machine learning was able to distinguish between ECH and CCH with high sensitivity and specificity. Furthermore, we identified differences between ECHb to ECHr, with steroid profiles influenced by cluster attack frequency in ECH, a pattern not observed in CCH.

### Episodic cluster headache

We identified a robust reduction in all mineralocorticoids in ECHb compared to controls. To further investigate this finding, we assessed the paired steroid profile of individuals in and out of bout and found lower mineralocorticoids and glucocorticoids in ECHb. Because these steroids are synthesized exclusively in the adrenal glands, this suggests that ECHb represents a state of lower adrenal steroid synthesis compared to ECHr [4]. These changes are unlikely to be genetic as ECHr displayed a similar steroid profile to controls, indicating these changes are also reversible. Given that glucocorticoids are used therapeutically in CH, it could be questioned whether our findings represent a physiological phenomenon or an iatrogenic effect. However, our participants were included more than 30 days after exogenous corticosteroid therapy, in line with clinical guidelines for corticosteroid assessment. We therefore consider these findings to represent physiological changes. The decreased cortisol/DHEAS ratio observed in ECHb suggests reduced adrenal gland capacity, which may contribute to the reduced steroid levels. Together, this data suggests a suppression of adrenal function in ECHb, relative to ECHr.

The cause of this reduced adrenal synthesis can be speculatively linked to evidence of hypothalamic-pituitary activation in the ictal period in ECHb [1]. It is a well-established hypothesis that the hypothalamus is activated during a cluster attack in ECHb [1]. We found that adrenal and gonadal androgens, as well as mineralocorticoids were inversely correlated with time from attack, meaning that steroid levels were higher the closer sampling occurred to a cluster attack. This suggests that the HPA and HPG axes are activated around the time of the cluster attack in ECHb. The gonadotropin FSH showed similar inverse correlations with time since last attack whereas LH was unchanged: FSH has a longer serum half-life (2–4 h) compared to LH (30 min), meaning it may represent a more sensitive marker of HPG activation than LH, potentially explaining the absence of LH changes in our study.

These findings are further strengthened through the observation that a higher attack frequency was associated with higher steroid levels. This proposed frequent peri-attack release of pituitary tropic hormones may induce desensitization of their receptors in the adrenal glands and hypothalamus. This is supported by previous studies demonstrating reduced ACTH and cortisol responses to pituitary stimulus in ECHb, providing a case for central suppression of adrenal function [5,6]. These mechanisms may also explain our findings of compensated hypogonadism in males with ECHb. Prolonged repeated peri-attack gonadotropin release may desensitize LH receptors in testicular Leydig cells, requiring higher LH levels to maintain adequate testosterone production.

Previous studies have assessed the levels of cortisol in ECH, with some studies finding increased cortisol and others finding unchanged cortisol levels [5,6,8–10]. Our present findings contradict these results. However, our cohort is substantially larger than these previous studies and steroids were quantified by LC-MS/MS rather than antibody-based assays [5,6,8–10]. Given the structural similarity of steroid hormones, LC-MS/MS ensures greater specificity and avoids the cross reactivity inherent to antibody-based assays, which can lead to overestimation of steroid concentrations [17].

### Chronic cluster headache

In CCH, we identified reduced 17-OH pregnenolone and DHEAS levels together with a higher cortisol/DHEAS ratio compared with controls. Reduced 17-OH pregnenolone and DHEAS is suggestive of impaired adrenal androgen synthesis, given that these steroids are primarily produced in the adrenal glands and are key intermediates in androgen biosynthesis [4]. This suggests that the compensated hypogonadism described in CCH may partly result from reduced adrenal androgen synthesis, leading to diminished availability of androgen precursors in the periphery [3]. Explanations for this could be ACTH insensitivity, zona reticularis atrophy, adrenal fatigue or that the adrenals preferentially synthesizing one steroid class over another (metabolic shuttle). Notably, steroid levels were not associated with attack frequency or proximity to sampling, in contrast to ECH, suggesting that cluster attacks in CCH may not be accompanied by the release of tropic hormones. We do, however demonstrate an increased cortisol/DHEAS ratio, an indicator of chronic stress and inflammation. Here, the adrenal gland favours synthesizing the catabolic glucocorticoids over the anabolic androgens, providing evidence for the metabolic shuttle.

Inflammation both influences and is influenced by steroid hormones; thus the circulating inflammatory milieu of CCH may influence its steroid phenotype. Interleukin-6 (IL-6), a putative regulator of adrenal steroid synthesis has been demonstrated to be elevated in this CCH cohort [15]. We found that serum IL-6 was inversely correlated with 17-OH pregnenolone and DHEAS, in keeping with a previous study on healthy individuals [18]. In addition, IL-6 has been demonstrated to directly suppress adrenal androgen synthesis, whereas IL-6 inhibition increases levels of 17-OH pregnenolone and DHEAS [16, 19]. Given this evidence, it is plausible that higher IL-6 in CCH contributes to adrenal androgen suppression, but this hypothesis requires further investigation.

The elevated cortisol/DHEAS ratio may have broader consequences, given that it has been suggested as a marker for chronic stress. A higher cortisol/DHEAS ratio is associated with a catabolic state and metabolic disturbances such as hypertension, hyperglycaemia and hypertriglyceridemia, suggesting that clinicians should be aware of a potential increased cardiovascular risk in CCH [20]. This is in line with observations of increased cardiovascular disease prevalence among the CH population [21, 22].

### Steroid differences between ECHb and CCH

We identified distinct steroid profiles across cluster headache states, characterized adrenal androgen suppression in CCH and corticosteroid suppression in ECHb (Supplemental Fig. 4).

This differential steroid profile can differentiate CCH from ECHb. Given this discriminatory capacity, steroid profiling could have diagnostic potential for the different cluster subtypes in the future. If validated, steroid profiling could have broad implications for stratification of treatment at the point of diagnosis or initiation of a new headache bout. It is important to establish if these findings hold in newly diagnosed CH.

Independent of their predictive value, the divergent steroid profiles and responses to cluster attacks suggest fundamental differences between CCH and ECH. The activation of the HPA and HPG axes observed in ECH but not in CCH suggests distinct underlying neurobiological mechanisms. Together with previous findings of distinct inflammatory profiles between CCH and ECH, these results point to a tantalizing insight: that CCH and ECH could represent distinct disease entities [15].

### Strengths and limitations

We present evidence from a large prospectively recruited cohort encompassing the cluster headache stages, providing a representative view of all the disease spectrum. Moreover, our paired analysis provides a dynamic insight into the physiology, preventing bias as individuals can act as their own control. Steroid differences were confirmed with age-adjusted z-scores, as well as controlling for age and sampling timing in linear regression models, strengthening confidence that our results are robust and represent biological effects rather than confounders. Clinical data was collected in a semi-structured interview conducted by trained headache specialists, ensuring high-quality clinical data. The use of gold standard LC-MS/MS ensured high specificity and sensitivity in the measurement of steroids.

We acknowledge that this study has some limitations. Firstly, ACTH and 11-oxygenated androgens were not measured, limiting interpretation of the underlying mechanisms of steroid dysregulation. Secondly, the study included only males, meaning that it is not applicable to females. Thirdly, recruitment from a tertiary headache centre may bias the cohort toward more severely affected individuals. The imbalance in smoking and medication exposure between the groups are potential confounders. In addition, our cluster cohorts were 13–14 years post-diagnosis, meaning we may see different profiles in those who are newly diagnosed. This could suggest our steroid profiles represent an established form of cluster headache.

The present study lends itself to future studies to enable us to fully understand the role of the hypothalamus and the observed differences between cluster headache states. Future studies should assess all hypothalamic releasing hormones and pituitary tropic hormones. This would be particularly important to assess with serial measurements in the pre- and post-attack period to establish the dynamics of the hypothalamic and pituitary hormones in the attack period.

## Conclusions

Overall, our findings suggest that adrenal gland dysfunction may underlie the disrupted circulating steroid concentrations in cluster headache, rather than gonadal dysfunction. Clear differences were observed between the steroid phenotypes of CCH and ECHb. These alterations are, however, likely secondary to other factors, rather than causal drivers of cluster headache attacks. Our findings support the notion that CCH and ECH may have distinct neurobiological underpinnings, despite presenting with similar clinical phenotypes. Future research is needed to understand the mechanisms driving these steroid differences and their role in the broader phenotype of cluster headache.

## Supplementary Information

Below is the link to the electronic supplementary material.

Supplementary Material 1

## Data Availability

The Danish Cluster Headache Biobank contains sensitive information and can consequently not be shared in full form according to Danish data protection law. De-identified data that underlie the results of this article can be shared upon reasonable request.

## Abbreviations

11DOC: 11-deoxycortisol
17OHPreg: 17-hydroxy pregnenolone
17OHP: 17-hydroxy progesterone
A4: Androstenedione
ALDO: Aldosterone
CH: Cluster headache
CCH: Chronic cluster headache
CORT: Corticosterone
DHEA: Dehydroepiandrosterone
DHEAS: Dehydroepiandrosterone- sulphate
DHT: Dihydrotestosterone
DOC: 11-deoxycorticosterone
E: Cortisone
ECH: Episodic cluster headache
ECHb: Episodic cluster headache bout
ECHr: Episodic cluster headache remission
E1S: Estrone-1-sulphate
F: Cortisol
FSH: Follicle-stimulating hormone
LC-MS/MS: Liquid chromatography- tandem mass-spectrometry
LH: Luteinizing hormone
PROG: Progesterone
T: Testosterone
