# Supplementary material for: Adrenal gland dysfunction in males with cluster headache
Source: J Headache Pain. 2026 May 13;27(1):176. doi: 10.1186/s10194-026-02386-z (PMC13348684; doi:10.1186/s10194-026-02386-z)
Supplement: Supplementary file 1 — Supplementary Material 1 [file 10194_2026_2386_MOESM1_ESM.docx]

**Supplemental material contents page**

 **Supplemental Figures**

Page 2: Supplemental Figure 1: Adrenal steroid synthesis

Page 3: Supplemental Figure 2: Full steroid profile in cluster headache, referring to figure 1

Page 4: Supplemental Figure 3: Age adjusted Z-scores for steroids in cluster headache

Page 5: Supplemental Figure 4: Hypothesis diagram

**Supplemental tables**

Page 6: Supplemental Table 1: Mass-spectrometry parameters

Page 7: Supplemental Table 2: Model for age and time of sampling adjustment

Page 8: Supplemental Table 3: Age Z-scores table

Page 9: Supplemental Table 4: Attacks in the past 24 hours ECHb.

Page 10: Supplemental Table 5: The effects of attack proximity and attack frequency on steroids in males with ECH in bout.

Page 11: Supplemental Table 6: Attacks in the past 24 hours CCH.

Page 12: Supplemental Table 7: The effects of attack proximity and attack frequency on steroids in males with CCH.

Supplemental Figures


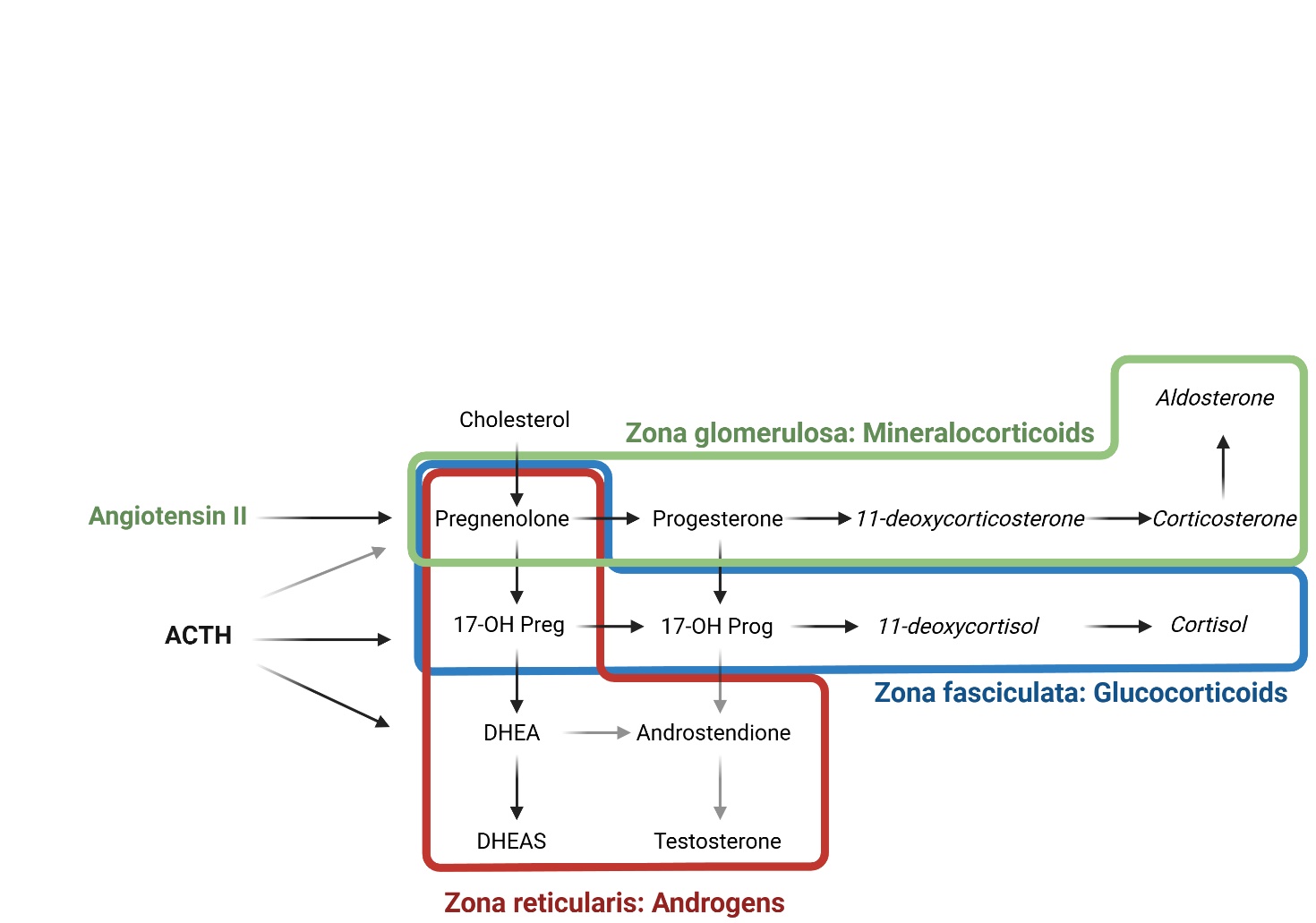


**Supplemental Figure 1: Adrenal steroid synthesis**

A schematic of adrenal steroid synthesis. The zona glomerulosa is under the influence of angiotensin II with a minor influence of ACTH, which stimulates the synthesis of pregnenolone and the mineralocorticoids. The zona fasciculata is under the influence of ACTH and synthesizes the glucocorticoids. The zona reticularis is under the influence of ACTH and synthesizes the adrenal androgens, note that androstenedione and testosterone are minor adrenal androgens and are not major contributors to the circulating androgen pool in males. Steroids in italics can only be synthesized in the adrenal glands. Faded arrow represent minor biosynthetic pathways or stimulus. ACTH = adrenocorticotropic hormone. Made in biorender.

**
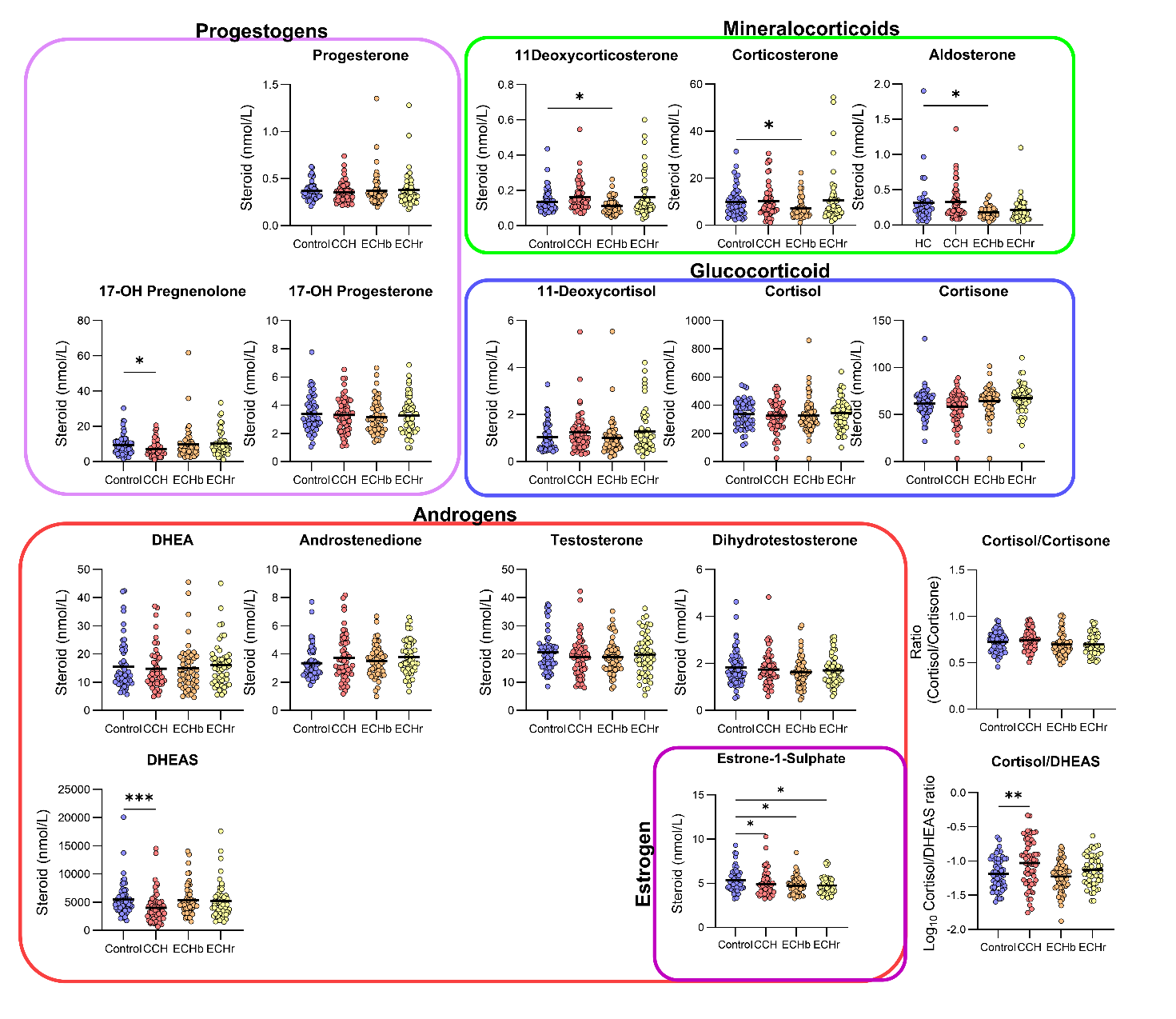
**

**Supplemental figure 2. Full steroid profile in cluster headache. Referring to figure 1**

The age and time of sampling adjusted steroid levels for each hormone assessed. Data presented as mean. One-way ANOVA with post-hoc Dunnett’s test on log_10_ adjusted data. *=P<0.05, **=P<0.01 and ***=P<0.001.


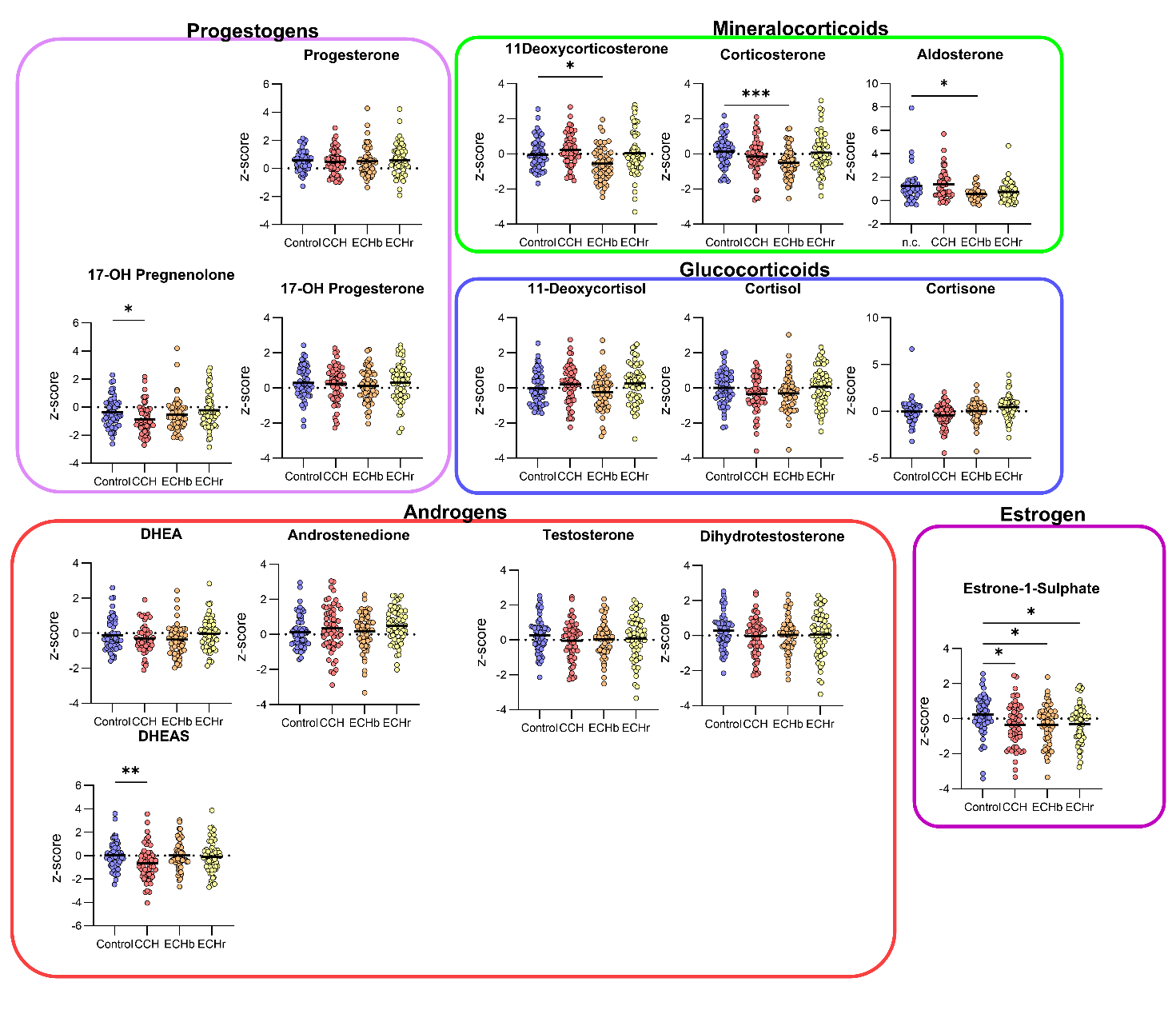


**Supplemental figure 3:** **Age adjusted Z-scores for steroids in cluster headache**

Age adjusted z-score for each steroid hormone assessed. Dotted line represents the mean for a given age. Data presented as mean. One-way ANOVA with post-hoc Dunns test. *=P<0.05, **=P<0.01 and ***=P<0.001.


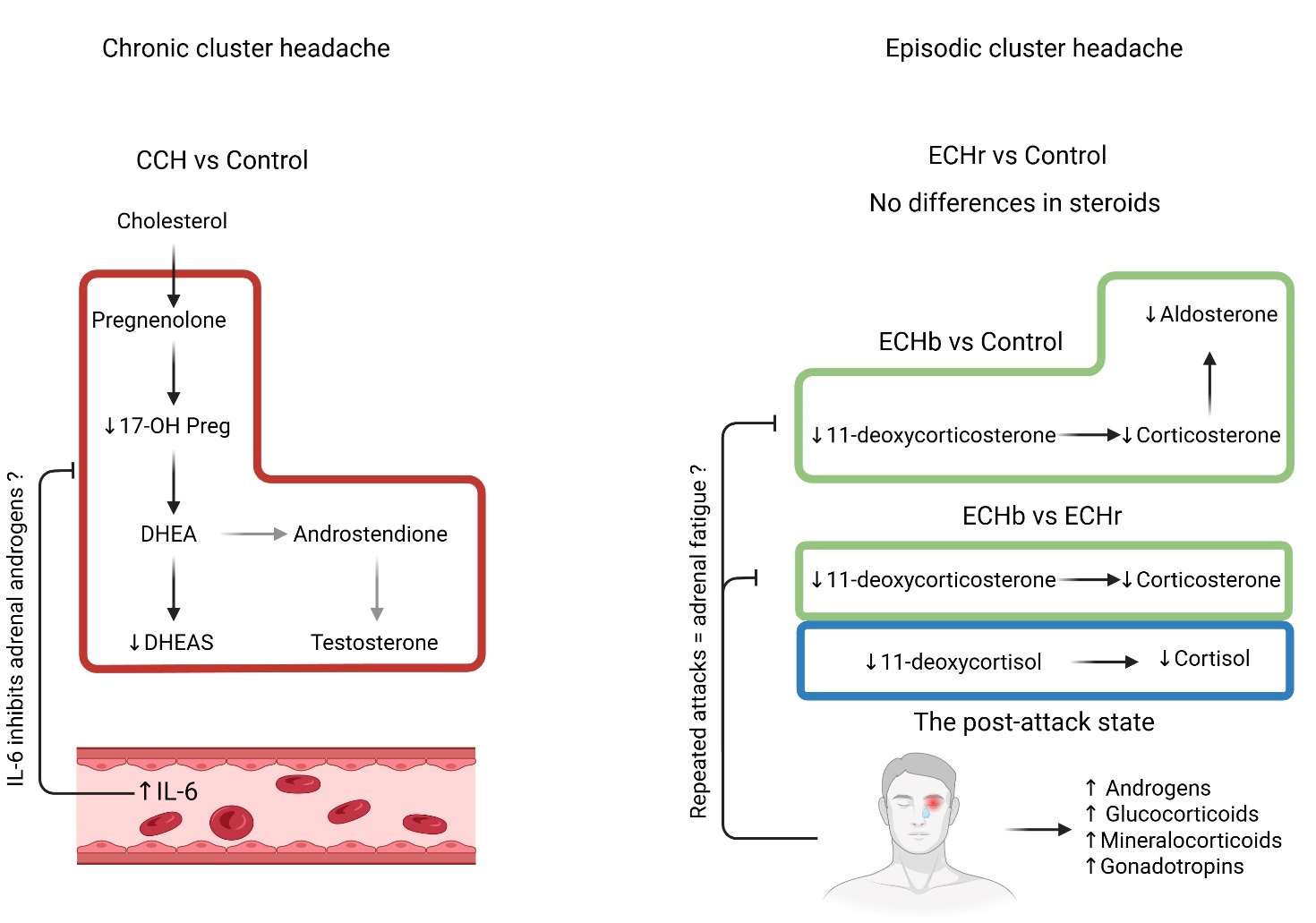


**Supplemental Figure 4: Hypothesis diagram**

In chronic cluster headache (CCH), we observe lower concentrations of adrenal androgens 17-hydroxy pregnenolone and DHEAS relative to controls, which could be suppressed by the higher interleukin-6 (IL-6) levels observed in CCH. In episodic cluster headache bout (ECHb) we find suppressed concentrations corticosteroids relative to controls and when the same patients are in remission (ECHr). This could be linked to apparent activation of the pituitary during the cluster attack, causing repetitive activation of the adrenal gland, desensitizing it to these signals. Made in biorender.

Supplemental Tables

| **Supplemental table 1 – Overview of steroid metabolites analysed by LC-MS/MS** | | | |  |
| --- | --- | --- | --- | --- |
| **Pathway** | **Steroid Metabolites** | **Abbreviation** | **LOD (nmol/L)** | **LOD (pmol/L)** |
| **Progestins** | Progesterone | PROG | 0.036 |  |
|  | 17-Hydroxypregnenolone | 17-OHPreg | 0.19 |  |
|  | 17-Hydroxyprogesterone | 17-OHP | 0.033 |  |
| **Glucocorticoids** | 11-Deoxycortisol | 11-DOC | 0.042 |  |
|  | 21-Deoxycortisol | 21-DOC | 0.035 |  |
|  | Cortisol | Cortisol | 5.57 |  |
|  | Cortisone | Cortisone | 0.64 |  |
| **Mineralocorticoids** | 11-Deoxycorticosterone | DOC | 0.029 |  |
|  | Corticosterone | CORT | 0.22 |  |
|  | Aldosterone | ALDO | 0.038 |  |
| **Androgens** | Dehydroepiandrosterone | DHEA | 4.4 |  |
|  | Dehydroepiandrosterone sulphate | DHEAS | 4.51 |  |
|  | Androstenedione | A4 | 0.031 |  |
|  | Testosterone | T | 0.031 |  |
|  | Dihydrotestosterone | DHT | 0.12 |  |
| **Oestrogens** | Estrone | E1 |  | 2.93 |
|  | Oestradiol | E2 |  | 4.04 |
|  | Estriol | E3 |  | 12.3 |
|  | Estrone sulphate | E1S | 0.025 |  |

Supplemental table 2: Model for age and time of sampling adjustment.

The age and time of sampling adjusted model for the log_10_ transformed steroid hormones. Significant values in **bold**.

| Steroid | **CCH**  β (SE), p-value | **ECHb**  β (SE), p-value | **ECHr**  β (SE), p-value | **Age**  β (SE), p-value | **Time of sampling**  β (SE), p-value |
| --- | --- | --- | --- | --- | --- |
| Progestogens | | | | | |
| PROG | -0.02(0.02), 0.35 | -0.01(0.02), 0.53 | -0.008(0.2), 0.74 | -0.002(0.0007), **0.005** | 0.006(0.006), 0.29 |
| 17OHPreg | -0.11(0.05), **0.02** | -0.01(0.05), 0.83 | 0.02(0.5), 0.67 | -0.009(0.001), **<0.0001** | -0.02(0.01), 0.07 |
| 17OHP | -0.01(0.03), 0.66 | -0.02(0.03), 0.40 | -0.01(0.03), 0.59 | -0.003(0.001), **0.001** | 0.001(0.007), 0.87 |
| Mineralocorticoids | | | | | |
| DOC | 0.06(0.03), 0.08 | -0.08(0.03), **0.03** | 0.01(0.03), 0.61 | 0.0009(0.001), 0.43 | -0.009(0.009), 0.27 |
| CORT | -0.02(0.05), 0.74 | -0.12(0.05), **0.02** | -0.01(0.05), 0.73 | -0.001(0.001), 0.50 | -0.05(0.001), **<0.0001** |
| ALDO | 0.04(0.06), 0.42 | -0.15(0.06), **0.019** | -0.12(0.06), **0.04** | -0.003(0.001), 0.11 | 0.01(0.01), 0.48 |
| Glucocorticoids | | | | | |
| 11DOC | 0.06(0.04), 0.21 | -0.03(0.04), 0.49 | 0.06(0.04), 0.20 | 0.004(0.001), **0.004** | -0.01(0.01), 0.28 |
| Cortisol | -0.03(0.03),  0.33 | -0.03(0.03), 0.37 | 0.006(0.03), 0.85 | 0.005(0.001), 0.62 | -0.02(0.008), **0.01** |
| Cortisone | -0.05(0.03), 0.12 | -0.0008(0.03), 0.97 | 0.03(0.03), 0.23 | -0.001(0.0009), 0.29 | -0.002(0.007), 0.78 |
| Androgens | | | | | |
| DHEA | -0.02(0.04), 0.55 | -0.02(0.04), 0.53 | 0.02(0.04), 0.59 | -0.008(0.001), **<0.0001** | -0.3(0.01), **0.002** |
| DHEAS | -0.18(0.04), **<0.0001** | -0.02(0.04), 0.58 | -0.04(0.04), 0.34 | -0.01(0.001), **<0.0001** | 0.02(0.01), 0.08 |
| A4 | 0.02(0.03), 0.43 | 0.01(0.03), 0.63 | 0.04(0.02), 0.11 | -0.003(0.0009), **0.00033** | -0.0008(0.007) |
| T | -0.04(0.02), 0.11 | -0.04(0.02), 0.15 | -0.03(0.02), 0.26 | -0.002(0.0009),  **0.003** | 0.004(0.007), 0.54 |
| DHT | -0.01(0.03), 0.56 | -0.04(0.03), 0.10 | -0.02(0.03), 0.51 | -0.0008(0.001), 0.39 | 0.004(0.007), 0.55 |
| Oestrogen | | | | | |
| E1S | -0.14(0.05), **0.009** | -0.14(0.05), **0.006** | -0.12(0.05), **0.01** | 0.0004(0.001), 0.79 | 0.01(0.01), 0.18 |
| Derivative measurements | | | | | |
| Cortisol/Cortisone | 0.01(0.02), 0.46 | -0.03(0.02), 0.18 | -0.03(0.02), 0.16 | 0.001(0.0006), **0.02** | -0.01(0.005), **0.0005** |
| Cortisol/DHEAS | 0.05(0.2), **<0.0001** | 0.002(0.1), 0.80 | 0.005(0.1), 0.60 | 0.002(0.0003), **<0.0001** | -0.007(0.002), **0.006** |

Abbreviations: PROG = progesterone, 17OHPreg = 17-hydroxy pregnenolone, 17OHP = 17-hydroxy progesterone, DOC = 11-deoxycorticosterone, CORT = corticosterone, ALDO = aldosterone, 11DOC = 11-deoxycortisol, DHEA = dehydroepiandrosterone, DHEAS = dehydroepiandrosterone- sulphate, A4 = androstenedione, T = testosterone, DHT = dihydrotestosterone and E1S = estrone-1-sulphate

| Steroid | Control (n=60) | CCH (n=59) | ECHb (n=58) | ECHr (n=59) |
| --- | --- | --- | --- | --- |
| Progestogens Z-score | | | | |
| Progesterone | 0.58 (0.73) | 0.45 (0.85) | 0.49 (1.00) | 0.58 (1.10) |
| 17-OH Pregnenolone | -0.36 (1.03) | -0.87 (1.04)* | -0.55 (1.13) | -0.22 (1.25) |
| 17-OH Progesterone | 0.28 (0.91) | 0.23 (1.02) | 0.11 (0.90) | 0.31 (1.07) |
| Mineralocorticoids Z-score | | | | |
| 11-deoxycorticosterone | -0.02 (0.85) | 0.24 (0.90) | -0.55 (0.94)* | 0.05 (1.26) |
| Corticosterone | 0.14 (0.85) | -0.14 (1.00) | -0.51 (0.83)*** | 0.07 (1.12) |
| Aldosterone^1^ | 1.23 (1.47) | 1.37 (1.22) | 0.58 (0.54)* | 0.73 (0.87) |
| Glucocorticoids Z-score | | | | |
| 11-deoxycortisol | -0.01 (0.94) | 0.19 (1.07) | -0.23 (0.99) | 0.25 (1.12) |
| Cortisol | 0.00 (0.94) | -0.36 (0.98) | -0.31 (1.02) | 0.08 (1.09) |
| Cortisone | -0.03 (1.25) | 0.42 (1.17) | 0.03 (1.08) | 0.45 (1.17) |
| Androgens Z-score | | | | |
| Dehydroepiandrosterone^2^ | -0.11 (0.90) | -0.31 (0.84) | -0.37 (0.90) | -0.02 (0.91) |
| DHEAS | 0.04 (1.14) | -0.66 (1.42)** | 0.01 (1.22) | -0.13 (1.35) |
| Androstenedione | 0.11 (0.92) | 0.35 (1.32) | 0.18 (1.05) | 0.49 (0.98) |
| Testosterone | 0.27 (0.97) | -0.04 (0.97) | 0.04 (0.98) | 0.07 (1.23) |
| Dihydrotestosterone | 0.27 (0.97) | -0.04 (1.08) | 0.04 (0.98) | 0.07 (1.23) |
| Oestrogen Z-score | | | | |
| Estrone-1-sulphate | 0.21 (1.12) | -0.34 (1.24)* | -0.34 (1.11)* | -0.29 (1.10)* |
|  |  |  |  |  |

Supplemental table 3: Z-scores.

The age adjusted Z-scores for steroids in controls, CCH, ECHb and ECHr. One-way ANOVA followed by post-hoc Dunns tests. Data presented as mean±SD. Delta and percentage is attack relative to no attack. *=P<0.05, **=P<0.01 and ***=P<0.001.

^1^ Due to values bellow lower limit of detection, for aldosterone N=40 for control, N=51 for CCH, N= 36 for ECHb and N=48 for ECHr. ^2^ For Due to values bellow lower limit of detection, for aldosterone N=58 for control, N=48 for CCH, N= 54 for ECHb and N=58 for ECHr.

Supplemental table 4: Attacks in the past 24 hours ECHb.

The steroid hormone profile in male ECHb patients who did not have an attack (n=23) vs those who had an attack (n=34) in the last 24-hours. T-tests performed on log_10_ age and time of sampling adjusted steroid values. Data presented as mean±SD. Delta and percentage is attack relative to no attack.

|  | No attack (n=23) | Attack (n=34) | Δ (%) | P-value |
| --- | --- | --- | --- | --- |
| Progestogens (nmol/l) | | | | |
| Progesterone | 0.35 (0.10) | 0.39 (0.21) | 0.04 (11%) | 0.51 |
| 17-OH Pregnenolone | 7.60 (3.02) | 11.08 (11.13) | 3.49 (46%) | 0.22 |
| 17-OH Progesterone | 3.19 (1.40) | 3.13 (0.96) | -0.06 (-2%) | 0.82 |
| Mineralocorticoids (nmol/l) | | | | |
| DOC | 0.09 (0.03) | 0.13 (0.06) | 0.04 (38%) | 0.04 |
| CORT | 7.28 (5.30) | 7.56 (4.83) | 0.28 (4%) | 0.81 |
| ALDO | 0.18 (0.071) | 0.186 (0.093) | 0.006 (3%) | 0.86 |
| Glucocorticoids (nmol/l) | | | | |
| 11-DOC | 0.79 (0.36) | 1.13 (0.94) | 0.34 (44%) | 0.07 |
| Cortisol | 329.45 (108.72) | 324.7 (132.37) | -4.75 (-1%) | 0.59 |
| Cortisone | 65.72 (15.24) | 63.184 (14.35) | -2.54 (-3.86%) | 0.43 |
| Androgens (nmol/l) | | | | |
| DHEA | 12.663 (4.862) | 16.483 (9.718) | 3.82 (30%) | 0.12 |
| DHEAS | 4755 (2484) | 5741 (2772) | 986 (21%) | 0.15 |
| Androstenedione | 3.10 (0.89) | 3.77 (1.16) | 0.68 (22%) | 0.04 |
| Testosterone | 16.73 (5.36) | 20.41 (5.73) | 3.67 (22%) | 0.01 |
| Dihydrotestosterone | 1.31 (0.49) | 1.83 (0.67) | 0.52 (40%) | 0.49 |
| Oestrogen (nmol/l) | | | | |
| Estrone-1-sulphate | 4.65 (1.07) | 4.79 (0.91) | 0.14 (3%) | 0.47 |
| Gonadotropins (ng/ml) | | | | |
| LH | 3.42 (1.67) | 3.77 (1.27) | 0.35 (10%) | 0.36 |
| FSH | 4.55 (2.08) | 6.21 (3.46) | 1.66 (36%) | 0.043 |
| Derivative measurements | | | | |
| Cortisol/cortisone | 5.07 (1.53) | 5.29 (1.92) | 0.22 (4.34%) | 0.78 |
| Cortisol/DHEAS | 0.08 (0.03) | 0.06 (0.03) | -0.02 (-20%) | 0.055 |

Abbreviations: DOC = 11-deoxycorticosterone, CORT = corticosterone, ALDO = aldosterone, 11DOC = 11-deoxycortisol, DHEA = dehydroepiandrosterone, DHEAS = dehydroepiandrosterone- sulphate LH = luteinizing hormone and FSH = follicle-stimulating hormone

Supplemental table 5: The effects of attack proximity and attack frequency on steroids in males with ECH in bout. Pearson’s correlation on log_10_ age and time of sampling adjusted steroid values.

|  | Time from last attack | | Attacks in the last 24 hours | | Attacks in the last week | |
| --- | --- | --- | --- | --- | --- | --- |
|  | Correlation | P-Value | Correlation | P-Value | Correlation | P-Value |
| Progestogens | | | | | | |
| PROG | -0.19 | 0.18 | 0.27 | 0.06 | 0.16 | 0.27 |
| 17OHPreg | -0.27 | 0.04 | 0.32 | 0.02 | 0.31 | 0.04 |
| 17OHP | -0.06 | 0.61 | -0.01 | 0.93 | -0.10 | 0.50 |
| Mineralocorticoids | | | | | | |
| DOC | -0.43 | 0.001 | 0.53 | <0.0001 | 0.52 | 0.0002 |
| CORT | -0.13 | 0.36 | 0.16 | 0.27 | 0.16 | 0.29 |
| ALDO | 0.02 | 0.89 | -0.15 | 0.41 | -0.24 | 0.19 |
|  | | | | | | |
| 11DOC | -0.34 | 0.01 | 0.44 | 0.002 | 0.42 | 0.003 |
| Cortisol | -0.01 | 0.97 | 0.15 | 0.28 | 0.15 | 0.30 |
| Cortisone | 0.09 | 0.53 | 0.09 | 0.55 | 0.11 | 0.44 |
| Androgens | | | | | | |
| DHEA | -0.28 | 0.04 | 0.27 | 0.07 | 0.23 | 0.13 |
| DHEAS | -0.09 | 0.53 | 0.22 | 0.13 | 0.25 | 0.09 |
| A4 | -0.30 | 0.03 | 0.40 | 0.004 | 0.40 | 0.005 |
| T | -0.43 | 0.001 | 0.41 | 0.003 | 0.30 | 0.04 |
| DHT | -0.39 | 0.003 | 0.34 | 0.02 | 0.20 | 0.18 |
| Oestrogen | | | | | | |
| ES1 | -0.23 | 0.10 | 0.29 | 0.04 | 0.33 | 0.02 |
| Gonadotropins | | | | | | |
| LH | -0.23 | 0.09 | 0.12 | 0.41 | 0.08 | 0.58 |
| FSH | -0.31 | 0.02 | 0.29 | 0.04 | 0.31 | 0.03 |
| Derivative measurements | | | | | | |
| Cortisol/cortisone | -0.14 | 0.32 | 0.12 | 0.42 | 0.07 | 0.63 |
| Cortisol/DHEAS | 0.11 | 0.42 | -0.10 | 0.48 | -0.12 | 0.42 |

Abbreviations: PROG = progesterone, 17OHPreg = 17-hydroxy pregnenolone, 17OHP = 17-hydroxy progesterone, DOC = 11-deoxycorticosterone, CORT = corticosterone, ALDO = aldosterone, 11DOC = 11-deoxycortisol, DHEA = dehydroepiandrosterone, DHEAS = dehydroepiandrosterone- sulphate, A4 = androstenedione, T = testosterone, DHT = dihydrotestosterone and E1S = estrone-1-sulphate, LH = luteinizing hormone and FSH = follicle-stimulating hormone

Supplemental table 6: Attacks in the past 24 hours CCH.

The steroid hormone profile in male CCH patients who did not have an attack (n=21) vs those who had an attack (n=38) in the last 24-hours. T-tests performed on log_10_ age and time of sampling adjusted steroid values. Data presented as mean±SD. Delta and percentage is attack relative to no attack.

| nmol/l | No attack (n=21) | Attack (n=38) | Δ (%) | P-value |
| --- | --- | --- | --- | --- |
| Progestogens (nmol/l) | | | | |
| Progesterone | 0.36 (0.10) | 0.35 (0.11) | -0.02( -4%) | 0.45 |
| 17-OH Pregnenolone | 6.68 (5.15) | 8.12 (5.45) | 1.44 (22%) | 0.20 |
| 17-OH Progesterone | 3.41 (0.96) | 3.23 (1.35) | -0.18 (-5%) | 0.30 |
| Mineralocorticoids (nmol/l) | | | | |
| DOC | 0.17 (0.11) | 0.16 (0.06) | -0.01 (-6%) | 0.90 |
| CORT | 9.65 (7.10) | 10.43 (7.22) | 0.78 (8%) | 0.57 |
| ALDO | 0.33 (0.19) | 0.33 (0.27) | 0.002 (1%) | 0.69 |
| Glucocorticoids (nmol/l) | | | | |
| 11-DOC | 1.11 (1.10) | 1.30 (0.66) | 0.19 (17%) | 0.11 |
| Cortisol | 311.69 (103.84) | 331.18 (106.59) | 19.45 (6%) | 0.47 |
| Cortisone | 58.12 (17.20) | 58.05 (15.10) | -0.07 (-0.1%) | 0.64 |
| Androgens (nmol/l) | | | | |
| DHEA | 13.73 (7.03) | 15.41 (8.22) | 1.68 (12%) | 0.52 |
| DHEAS | 4027 (3286) | 4001 (2353) | -26.92 (-0.7%) | 0.54 |
| Androstenedione | 3.51 (1.44) | 3.83 (1.74) | 0.32 (9%) | 0.49 |
| Testosterone | 19.73 (7.53) | 18.62 (6.44) | -1.11 (-6%) | 0.74 |
| Dihydrotestosterone | 1.77 (0.72) | 1.72 (0.70) | -0.05 (-3%) | 0.94 |
| Oestrogen (nmol/l) | | | | |
| Estrone-1-sulphate | 4.77 (1.53) | 4.94 (1.28) | 0.17 (4%) | 0.54 |
| Gonadotropins (ng/ml) | | | | |
| LH | 4.54 (2.34) | 4.68 (2.90) | 0.14 (3%) | 0.84 |
| FSH | 7.29 (4.40) | 7.04 (5.85) | -0.25 (-3%) | 0.86 |
| Derivative measurements | | | | |
| Cortisol/Cortisone | 5.53 (1.37) | 5.78 (1.56) | 0.24 (4%) | 0.60 |
| Cortisol/DHEAS | 0.12 (0.08) | 0.13 (0.11) | 0.006 (5%) | 0.88 |

Abbreviations: DOC = 11-deoxycorticosterone, CORT = corticosterone, ALDO = aldosterone, 11DOC = 11-deoxycortisol, DHEA = dehydroepiandrosterone, DHEAS = dehydroepiandrosterone- sulphate LH = luteinizing hormone and FSH = follicle-stimulating hormone.

Supplemental table 7: The effects of attack proximity and attack frequency on steroids in males with CCH. Pearson’s correlation on log_10_ age and time of sampling adjusted steroid values.

|  | Time to last attack | | Attacks in the last 24 hours | | Attacks in the last week | |
| --- | --- | --- | --- | --- | --- | --- |
|  | Correlation | P-Value | Correlation | P-Value | Correlation | P-Value |
| Progestogens | | | | | | |
| PROG | 0.06 | 0.66 | -0.04 | 0.75 | -0.05 | 0.72 |
| 17OHPreg | -0.14 | 0.28 | 0.19 | 0.15 | 0.24 | 0.06 |
| 17OHP | 0.05 | 0.68 | -0.13 | 0.34 | -0.02 | 0.86 |
| Mineralocorticoids | | | | | | |
| DOC | -0.01 | 0.94 | 0.29 | 0.03 | 0.06 | 0.67 |
| CORT | -0.14 | 0.28 | 0.21 | 0.10 | 0.22 | 0.10 |
| ALDO | 0.16 | 0.25 | 0.07 | 0.60 | -0.11 | 0.43 |
| Glucocorticoids | | | | | | |
| 11DOC | -0.23 | 0.08 | 0.12 | 0.36 | 0.29 | 0.03 |
| Cortisol | -0.14 | 0.31 | 0.22 | 0.10 | 0.18 | 0.19 |
| Cortisone | -0.05 | 0.71 | -0.12 | 0.41 | 0.02 | 0.88 |
| Androgens | | | | | | |
| DHEA | -0.07 | 0.63 | 0.04 | 0.77 | 0.10 | 0.49 |
| DHEAS | -0.11 | 0.39 | 0.07 | 0.60 | 0.08 | 0.57 |
| A4 | -0.07 | 0.58 | 0.19 | 0.15 | 0.17 | 0.20 |
| T | 0.01 | 0.95 | -0.09 | 0.49 | -0.04 | 0.74 |
| DHT | -0.05 | 0.73 | -0.07 | 0.61 | 0.00 | 0.99 |
| Oestrogen | | | | | | |
| ES1 | 0.00 | 0.99 | -0.11 | 0.40 | -0.13 | 0.34 |
| Gonadotropins | | | | | | |
| LH | 0.00 | 0.25 | -0.16 | 0.23 | 0.39 | 0.39 |
| FSH | 0.01 | 0.88 | -0.16 | 0.23 | 0.16 | 0.16 |
| Derivative measurements | | | | | | |
| Cortisol/cortisone | -0.17 | 0.21 | 0.27 | 0.04 | 0.30 | 0.02 |
| Cortisol/DHEAS | 0.02 | 0.89 | 0.06 | 0.65 | 0.09 | 0.51 |

Abbreviations: PROG = progesterone, 17OHPreg = 17-hydroxy pregnenolone, 17OHP = 17-hydroxy progesterone, DOC = 11-deoxycorticosterone, CORT = corticosterone, ALDO = aldosterone, 11DOC = 11-deoxycortisol, DHEA = dehydroepiandrosterone, DHEAS = dehydroepiandrosterone- sulphate, A4 = androstenedione, T = testosterone, DHT = dihydrotestosterone and E1S = estrone-1-sulphate, LH = luteinizing hormone and FSH = follicle-stimulating hormone.
